# Representing living architecture through skeleton reconstruction from point clouds

**DOI:** 10.1038/s41598-022-05194-y

**Published:** 2022-01-28

**Authors:** Wilfrid Middleton, Qiguan Shu, Ferdinand Ludwig

**Affiliations:** Green Technologies in Landscape Architecture, Technical University of Munich TUM, 80333 Munich, Germany

**Keywords:** Civil engineering, Plant sciences, Computer science

## Abstract

Living architecture, changing in structure with annual growth, requires precise, regular characterisation. However, its geometric irregularity and topological complexity make documentation using traditional methods difficult and presents challenges in creating useful models for mechanical and physiological analyses. Two kinds of living architecture are examined: historic living root bridges grown in Meghalaya, India, and contemporary ‘Baubotanik’ structures designed and grown in Germany. These structures exhibit common features, in particular network-like structures of varying complexity that result from inosculations between shoots or roots. As an answer to this modelling challenge, we present the first extensive documentation of living architecture using photogrammetry and a subsequent skeleton extraction workflow that solves two problems related to the anastomoses and varying nearby elements specific to living architecture. Photogrammetry was used as a low cost method, supplying detailed point clouds of the structures’ visible surfaces. A workflow based on voxel-thinning (using deletion templates and adjusted p-simplicity criteria) provides efficient, accurate skeletons. A volume reconstruction method is derived from the thinning process. The workflow is assessed on seven characteristics beneficial in representing living architecture in comparison with alternative skeleton extraction methods. The resulting models are ready for use in analytical tools, necessary for functional, responsible design.

## Introduction

**Figure 1:**
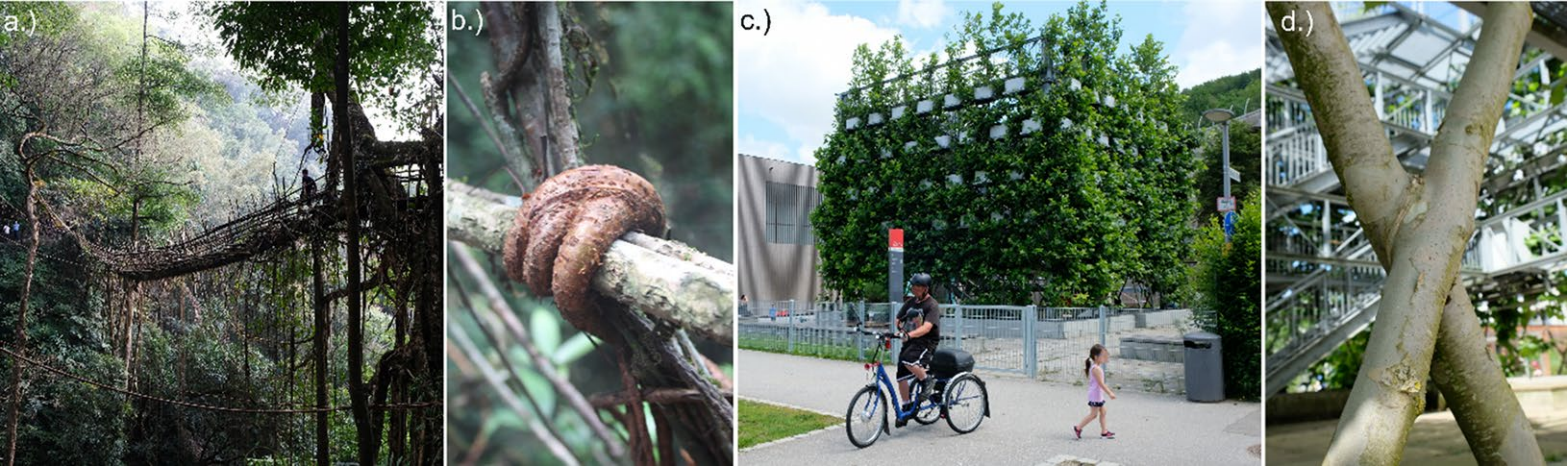
Living architecture exhibits points of interest on a range of scales: (**a**) a 53 m long living root bridge (photo: W. Middleton), (**b**) details in bridges are at the cm scale (W. Middleton), (**c**) the Nagold Plane Tree Cube, a contemporary Baubotanik example (F. Ludwig), (**d**) an inosculation in the Plane Tree Cube (F. Ludwig).

Living architecture, created by shaping and merging trees encompasses vernacular and professionally designed structures in temperate, subtropical and tropical settings. It has been adopted in recent decades by architects and designers worldwide to address aspects of urban ecology and climate change adaptation (e.g. by Arbona et al.^1^). Historic examples range from German Tanzlinden^2^ and Meghalaya’s living root bridges^3^, which have recently become famous worldwide, to simple rural practices such as hedge laying (e.g. in the UK)^4^. This study focuses on Meghalaya’s living root bridges (LRBs) and contemporary ‘Baubotanik’ structures. In their pilot study Ludwig et al.^3^ describe 75 of Meghalaya’s LRBs, as well as ladders, platforms and pathways, which form transport networks and cultural heritage sites for rural and urban communities^5^. LRBs are grown from *Ficus elastica* aerial roots and are mainly situated on steep slopes in deep valleys and dense forests. The bridges are between 2 and 53 m long, (Fig. 1a) with details (e.g. inosculations and bark features) at the centimetre scale (Fig. 1b). Structurally important roots in LRBs have circular, elliptical and ‘inverted-T’ cross-sections^3^, like subterranean roots in other trees^6^.

The German neologism Baubotanik describes a contemporary approach that utilises state-of-the-art methods to integrate living tree growth into building design (Fig. 1c). It is defined as a form of architecture in which structures are created through the interaction of technical and grown elements by manipulating the growth of trees or their parts, joining them with each other and connecting them with non-living components in such a way that they merge into a botanical-technical entity (Fig. 1d)^7, 8^. Growth manipulation and induced inosculation are central to both Meghalaya’s LRBs and contemporary Baubotanik structures, forming anastomotic networks. Through the interaction of different tree species with local environmental conditions, varying manipulation techniques and years or decades of growth processes, diverse and often highly complex topologies emerge.

By using growing organisms as integral parts of functional structures, living architecture holds great potential for environmentally sound and future-oriented building designs. These buildings bring together two distant fields of analysis: mechanics and growth. Both require a structural model that is topologically continuous and accurate and preserves element geometric features such as thickness, curvature and length. Godin et al.^9^ describes how geometry and topology inform plant growth, and their documentation provides realistic physiological models of growth and senescence. When considering plant biomechanics, beam theory provides a useful foundation^10^. Tree topology^11^ and element shape^12^ are vital in determining mechanical properties. But, as described by^13^, the complexity of tree structural forms is addressed in the literature only to a limited extend. This is partly because most authors have focused on conifer stands in the forestry industry and partly due to historical limits to data acquisition, before the recent availability of detailed point clouds (PCs). In particular, anastomoses are not commonly considered in tree skeleton models. The research gap that this reveals is addressed by the present study—the documentation of living architecture (in particular LRBs) in 3D detail and the provision of data structures that allow for structural and physiological analyses of historic and designed living architecture. In pursuit of this, we answer two questions. Firstly, can living architecture be documented in sufficient detail with off-the-shelf (OTS) equipment? Secondly, can a skeleton, with the capacity for volume reconstruction, be developed from PCs of living architecture?

In order to explain our choice of methods, the state of the art of PC data acquisition and skeleton extraction are discussed below with respect to the specific challenges of living architecture. Next, we explain the samples we investigate, the methods we apply and constituent steps of our workflow, and seven criteria on which skeletons of living architecture specimens should be assessed. As results of our study we present selected photogrammetric PCs of LRBs and example skeletons and reconstructed volumes of representative samples resulting from our workflow. We then assess the workflow and resulting skeletons with respect to the seven characteristics, in comparison with two other skeleton extraction algorithms. Finally, the workflow’s wider application in and beyond living architecture is discussed.

### State of the art of PC data acquisition and skeleton extraction

Large datasets are needed to fully describe the complex shapes present in living structures at the range of scales described above. Terrestrial laser scanners (TLS) and close-range photogrammetry (CRP) document the visible surfaces of objects. They are used in the field of architectural heritage, where large buildings are documented, along with small scale details marking unique features, historic techniques and ongoing decay. Fassi et al.^14^ show CRP and TLS are useful at scales similar to this study. The use of CRP in combination with other techniques has come a long way: Yilmaz et al.^15^ track fire damage of a historic building by combining photogrammetry and basic measurements, while recent studies have focused on TLS integration^16^, combined drone- and terrestrial CRP^17^ and specific downstream methods such as for construction sites and (H)BIM^16, 18^.

CRP and TLS are also compared in forestry and plant science. Many of these studies focus on automating the measurement of diameter at breast height (DBH), a common measure that allows inference of stand make-up. Single-tree surveys^19^ show better precision than multi-tree surveys^20^. Surový et al.^19^ show that five overlapping cameras are needed for good survey results, while more than eight is unnecessary for documenting forest trees. Forsman et al.^20^ show a 5-camera CRP rig produces inferior results to a TLS survey. Liang et al.^21^ find that handheld consumer cameras can provide photogrammetric PCs of similar accuracy to those resulting from TLS data in a forest stand of approximately 30 × 30 m. Mokroš et al.^22^ find photogrammetry suitable for stem reconstruction within stands. Hanke and Moser^23^ document a Tanzlinde tree using photogrammetry, though the extracted branching structure is significantly less complicated than the topologies of other living architecture mentioned above. Branches and roots are significantly more difficult to document than stems, mainly due to occlusions. Yoshinoa and Okardab^24^ recreate a Melaleuca specimen by informing a simulation model with photogrammetry-derived growth parameters. Changes in living architecture, including growth, senescence, epiphyte presence, and maintenance require more regular documentation than in heritage architecture^5^. Therefore, a tool that can be used regularly by the communities who grow and own living architecture, is easy to transport, is low cost, and requires little training, is preferred. Of the reviewed survey techniques, photogrammetry can provide relatively accurate data at a relatively low cost, with minimal training and lightweight tools. Therefore, CRP was identified as the most suitable method for acquiring the PCs that form the basis of this study.

Generating geometric-topological models of complex structures is a significant challenge. While in simple structures a small number of accurate data points can be collected (e.g. using tacheometry) to represent edges and corners onto which shape primaries are mapped, in complex structures (e.g. an irregular curve in a branch) simple curves and surfaces cannot be easily interpolated and edges and corners are not clear. This challenge is present, for example, in heritage documentation^14,25^. The shape primaries useful in mechanical and physiological tree models are typically 1D elements connected at branching and joining points. A wide range of studies reconstruct tree stems^26^, branches^27^ and whole-tree structures^24^ from a variety of LiDAR and photogrammetric PCs. Branches and roots that are essentially elliptic or circular in cross-section lend themselves well to skeletonisation, which produces a data-light model. Tagliasacchi et al.^28^ discuss the variety of skeletonisation methods available, categorising them by dimensionality and input spatial data. In particular, 1D-curve skeletons provide thin, centred structures that preserve tubular shapes (e.g. branches), well. Cornea et al.^29^ compare the main classes of 1D-curve skeletons and Bucksch et al.^30^ categorise 1D skeletonisation methods into five groups: geometric, clustering, graph reduction, medial axis, and morphological methods. Geometric methods^31^, such as Wang’s^32^ minimum spanning tree (MST) can process incomplete clouds, producing realistic but potentially false topologies. This causes particular problems for the anastomotic networks common in living architecture. Similarly, clustering methods, such as Xu’s^33^ can produce false topologies in detailed parts of PCs. Bucksch and Lindenbergh’s ‘graph-reduction’ method^34^ overlays an octree graph on the PC. It is computationally efficient and can represent topology well when the model’s voxels are large enough to cover gaps and noise in the PC but does not guarantee connectedness. Medial axis methods, such as that presented by Huang et al.^35^, effectively extract tree skeletons from relatively complete clouds^36,37^. However, two issues arise in application to network-like structures in living architecture. Firstly, the equal sized local neighbourhoods used throughout the PC require separate elements to be of roughly similar sizes or relatively distant from one another. When a small element is near a large one, the local attraction neighbourhood of points within the large element can engulf the small one. Secondly, by identifying separate elements, the method does not guarantee topology preservation (continuity between elements is only later applied, see bridge points in Huang et al.^35^). Both problems can be avoided using voxel-thinning (classified as a morphological method by Bucksch et al.^30^). Saha et al.^38^ describe two kinds of voxel-thinning: parallel thinning^39, 40^ and sequential thinning^41, 42^, of which the latter group is shown to preserve topology and provide 1-voxel-thick skeletons. As topology preservation is of central importance, sequential voxel-thinning is the most suitable skeletonisation method for living architecture—the method presented here draws on previous findings in this field.

Branches, stems and roots are axial elements with typically approximately circular, elliptical or other simple cross-sections^3^. Element cross-sectional shape and size inform mechanical and physiological models. Therefore, the skeletonisation process should preserve enough information to reconstruct the object’s volume accurately. Various methods for this have been documented: comparison of the skeleton with the original voxels^43^, cylinder fitting to the original PC^44^, and finding the radii and centres of maximum balls (CMB) in the voxel object^45^. Here, we define a new method that reconstructs the object volume based on information captured during the voxel-thinning process, avoiding reliance on reference to the original PC for comparison or fitting.

## Methods

### Photogrammetric surveys

This study is concerned with specimens with generally visible elements. Four representative samples are used to show the skeletonisation workflow below: two small-scale inosculated joints of different complexity and two large-scale structures. The first inosculated joint is a topologically relatively simple specimen, 50 cm long, from a pair of 7-year-old *Platanus hispanica* trees from a Baubotanik test field (hereinafter referred to as the Baubotanik joint). The second inosculated joint is a 1 m long part of a Living Root Ladder near Mawshun village in East Khasi Hills district, of unknown age (hereinafter referred to as the Ficus joint). The large scale structures are the Freiburg pavilion and Wah Koh La bridge. The Freiburg pavilion is a quite young (planted in 2017) and relatively regular structure. The 32 London Plane trees (*Platanus hispanica*) are planted in an oval with 10 m major diameter, 6.5 m minor diameter. The trees are grafted together at two points per tree and connected to a steel ring at the top, which is also supported by six vertical steel poles. A textile roof will be added in due course. Wah Koh La bridge is a two-span bridge across the seasonal Koh La river between Myntheng and Ramdait villages in East Khasi Hills district. The western span, the subject of this study, is 15.4 m long, grows between two trees (on the western bank a river island) and consists of several long roots forming a footway and handrails with many roots intergrown between them, similar to a simple suspension footbridge. It is thought to be 100–200 years old^3^, but individual root ages are unknown. Additionally, sections of individual roots from other LRBs are used to assess the volume reconstruction of elements with approximately elliptical cross-sections (hereinafter referred to as elliptical root samples).

In March 2018 and March 2019, 11 photogrammetric surveys were conducted for ten LRBs as well as five surveys of small details of other living architecture, partially described in Middleton et al.^46^. All bridges were scaled by the basic measurements described by Ludwig et al.^3^ Additionally, some bridges were scaled with diverse other methods. In November 2019 surveys of three individual Baubotanik inosculated joints and of the Freiburg pavilion were conducted. The surveys were performed using OTS cameras. For each structure only one camera was used. Logistical problems resulted in the use of two different DSLRs: a 12MP Canon EOS 450D DSLR with an APS-C sensor and an EF-S 18 mm f/3.5–5.6 lens for the 11 LRB surveys (including the elliptical root samples) and the five small detail surveys; a 24MP Fuji XT 20 DSLR (APS-C sensor; 18 mm, f/2.8–4 lens) for the Baubotanik joints and Ficus joint. A DJI Mavic 2 Pro drone with a 20MP Hasselblad L1D-20C camera (1″ sensor; 28 mm, f/2.8–f/11 lens) was used for the Freiburg pavilion. The number of photos varied between structures: longer bridges with more accessible angles were captured by more photos. The bridge models ranged from 121 to 1639 photos (mean 701, median 526). The detail and joint surveys used 58 to 150 photos (mean 104, median 97). Agisoft Metashape standard edition^47^ was used for all photogrammetric reconstructions using a Lenovo Thinkpad t470s (i5-7200U, 20 GB RAM). CloudCompare^48^ was used for basic orientation and trimming, as well as the scaling.

A range of scaling methods were used. Six LRBs were scaled to the basic measurements described by Ludwig et al.^3^. One LRB, in addition to these basic measurements, was scaled using five 127 mm square markers spaced on the deck. It was surveyed twice (in 2018 and 2019)—the resulting PCs were compared for distortion resulting from photogrammetric reconstruction. The PCs are aligned at eight points, then the distances from the less populated PC (2019) to the nearest points on the more populated PC were measured. For two other bridges and the pavilion, multiple measurement points provided scaling and a measure of distortion. 12 and 15 measurement points were marked with 2 cm-wide tape respectively. 21 and 47 measurements between these points were made using a Leica Disto D2 handheld laser. In the pavilion, 16 measurements were made with a tape measure between the bases of eight scaffolding poles. Wah Koh La bridge was measured using 13 element circumference measurements. The Ficus and Baubotanik joints were only measured by one length and one circumference each at an accuracy of 10 mm.

### Skeletonisation workflow

The semi-automated process for extracting shape-preserving skeletons from PCs in this study involves nine steps, written in C++ using the Point Cloud Library (PCL)^49^. All steps were developed and run on an Intel i7 (2.8 GHz 16 GB RAM). Steps 1–8 are sequential and perform the basic skeletonisation. Step 9 is the volume reconstruction utilising shape data and is defined for circular (9a) and elliptical (9b) cross-sections. The steps are detailed below.

**1. Orientation:** eigenvectors are defined for the PC using principal component analysis^50^. This is used to orient the cloud’s longest axis in the vertical direction, reducing the void voxel space.

**2. Voxelisation:** using the PCL^49^, an octree is formed, splitting each voxel into 8 sub-voxels at each depth level. Voxels inscribing points are defined as ‘object’ voxels; all others are ‘void’ voxels. Voxel length is manually defined, resulting in a specific octree depth. Voxel size is determined in part by the PC density—the gaps between points must be covered by the voxels (i.e. minimum of one point per voxel). Areas of low point density (or small surface holes) can be covered with large voxels; traded for lower precision in areas of high density. Large voxels can also help avoid distortions caused by noise or poor sampling. The octree depths used for the Ficus joint, Baubotanik joint, Freiburg pavilion, and Wah Koh La bridge are seven, eight, nine, and eight respectively.

**3. Cartesian coordinate conversion:** as steps 4, 6 & 7 involve operations in the cardinal directions, Cartesian coordinates are needed. Functions from the octree module of the PCL library^49^ ascribe Cartesian coordinates to each point in the octree.

**Figure 2:**
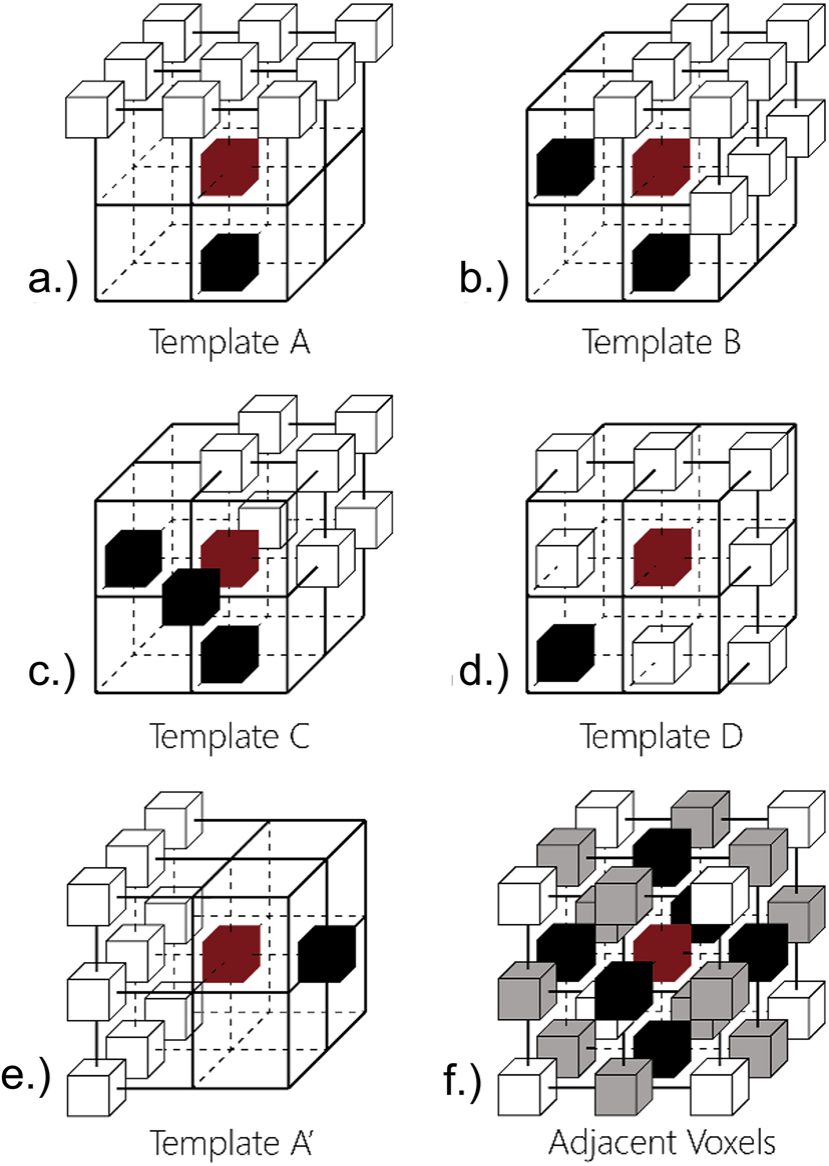
(**a**–**d**) show the four base template types used in step 7. One variant of template A is shown in (**e**). Each template assesses the 26 neighbour voxels in a 3 × 3 × 3 space around a candidate (red), discriminating between void voxels (white) and object voxels (black). (**f**) shows the 26-neighbourhood of the red voxel, with face-, edge-, and corner-connected voxels in black, grey and white respectively.

**4. Voxel denoising:** the noise present in the PC, voxelised in step 2, must be deleted to avoid erroneous topologies. A voxel can be 6-, 18- and 26-connected to other voxels, defined by the neighbourhood shown in Fig. 2f. This denoising step deletes any object voxels that are not 6-connected to the main object body (the largest connected group of object voxels). A 26-connectedness check was trialled, but was found to leave too many ‘loose ends’, which were unhelpfully represented in the final skeleton. Palágyi and Kuba^42^ show the impact of such noise on the final skeleton. While a more aggressive 6-connectedness check can lead to holes in the voxel surface (where a surface runs through 18- or 26- but not 6-connected neighbouring voxels), this appears to be relatively manageable (step 5).

**5. Surface filling:** PCs can exhibit gaps due to self-occlusion or poor sampling. Upon voxelisation, these gaps can translate into one or more voxels missing from an otherwise continuous voxel surface. Such holes misrepresent the object topology. They are filled manually. For a review of automated hole-filling processes, see Attene et al.^51^. Once occluded positions have been filled, the open ends of the branches that continue beyond the model space are also filled.

**6. Internal space filling:** the internal spaces are filled. Due to its applicability to complex models (with cavities or intertwining elements), a continuity-check was developed. Void voxels are assumed to be either external, in a single connected group; or internal, in one or more connected groups. Based on this, all voids that are not 6-connected to the external void are filled. This assumption does not allow for voids internal to the main body, though these are not captured by photogrammetric surveys.

**Figure 3:**
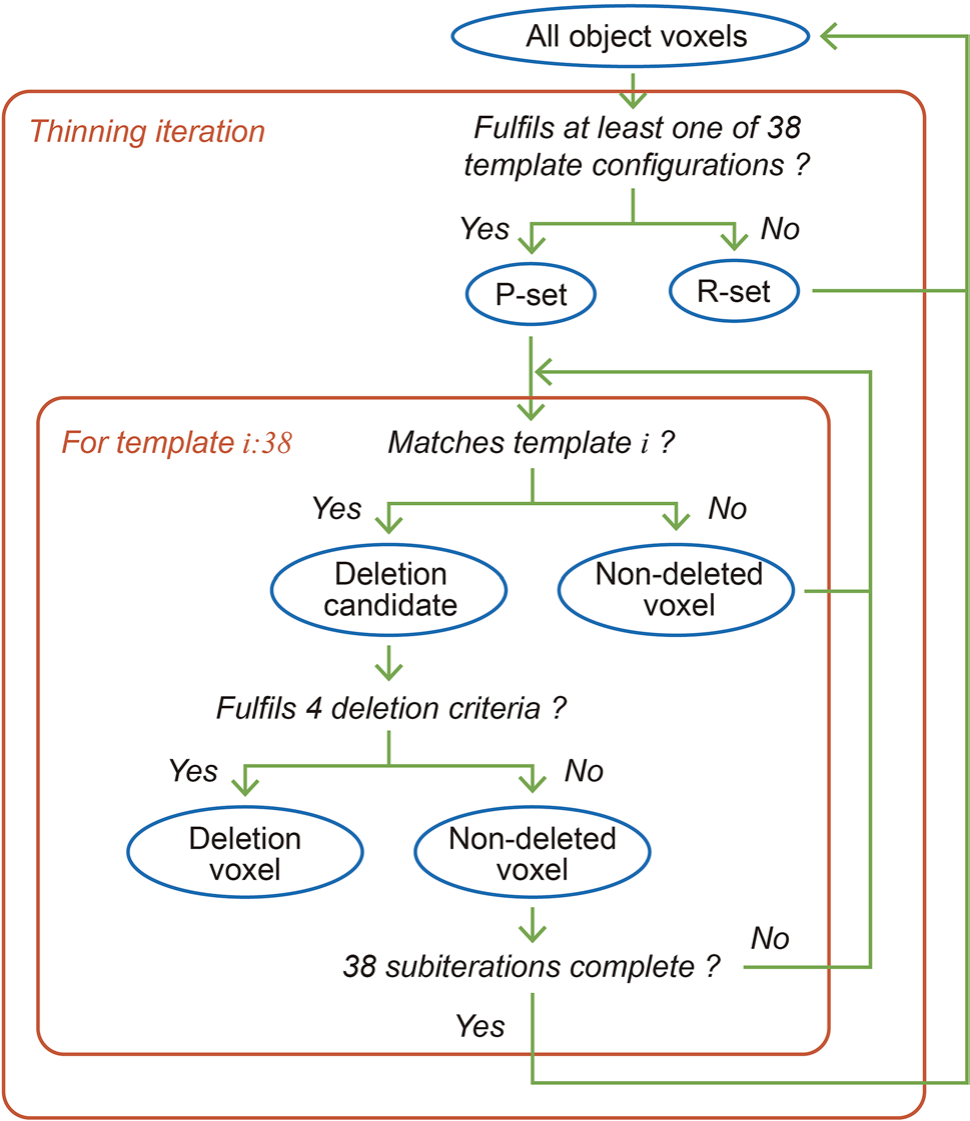
The iterative thinning in step 7.

**7. Voxel thinning:** a thin (1-voxel-thick) skeleton is extracted by iterative thinning. During thinning, object voxels are turned to void voxels (herein referred to as deletion). To do this She et al.^41^ adapt templates from Palágyi and Kuba^42^, making them more simple (avoiding Palágyi and Kuba’s “either/or” points). Four base templates (shown in Fig. 2a–d) can be rotated to make 6, 12, 8, and 12 unique configurations respectively (compare Fig. 2a, e). She et al.^41^ combine these 38 configurations with two deletion criteria that preserve topology. However, that method does not preserve curve-end voxels (object voxels with only one 26-adjascent object voxel), which represent branches that do not reach the space boundary. The method presented here combines the templates of^41^ with an adjustment to the P-simple deletion criteria proposed by Lohou and Bertrand^52^. Each object voxel is compared against the 38 configurations and all fulfilling voxels are checked for adjusted P-simplicity^52^—they are deleted if they meet the four criteria or returned to the set of all object voxels for the next thinning iteration if they don’t. An n-connected component consists of voxels linked in a chain by n-adjacency. As described by Palágyi and Kuba^42^, the order of directional thinning impacts the position of the remaining object. In order to provide a well-centred skeleton, we apply all directions of template A, followed by template B, C and D, rather than applying all templates in one direction then moving to the next direction. Figure 3 shows the process. The four deletion criteria for an object voxel are as follows:

For an object voxel *x* that fits at least one of the 38 templates, examining the 3 × 3 × 3 neighbourhood of *x*:

- All 26-adjacent object voxels must form a single, 26-connected component
- All 18-adjacent void voxels must form a single, 6-connected component
- For any 26-adjacent object voxel considered for deletion *y*, there exists another object voxel *z* that is 26-adjacent to both *y* and *x*. If no *y* exists, the criterion is satisfied.
- For any 6-adjacent object voxels considered for deletion (denoted y), there exist two void voxels (z and t) that form a unit square with *x* and *y*—each of *x,y,z* and *t* are 6-adjacent to two others. If no *y* exists, criterion d is satisfied.

As has been discussed elsewhere^38^, criteria (a) and (b) ensure that object chains are not broken and cavities are not connected, respectively. Criteria (c) and (d) protect curve ends from deletion. Where curve ends meet the boundary of the modelling space they do not fulfil any templates and are preserved. These criteria are adapted from Lohou and Bertrand^52^. Criterion (c) was changed to attain 1-voxel-thick skeletons: that study defines *z* as a non-deletable object voxel whereas here it must simply be an object voxel.

**8. 1D curve conversion (segmentation):** a 1D curve, fit between voxel centroids, provides the necessary topological connectivity between discrete voxels to form ‘branch’ elements, rather than the discrete unconnected voxels points^9^. Object voxels with three or more 26-neighbourhood object voxels are considered joint voxels, providing connectivity between elements. Busier junctions exhibit more than one joint voxel. In such cases, the voxel closest to the centroid of the group is chosen as the true joint, and others are described as associated joint voxels.

**9. Iteration of exposure volume reconstruction:** the 3D volume is reconstructed from the 1D skeleton by counting the iteration on which faces of the skeleton voxels are exposed and building out 2D circular or elliptical cross-sections from each skeleton voxel with radii proportionate to the iteration numbers. In a 1-voxel-thick skeleton, all voxels have a minimum of four exposed faces. The iteration on which the nth face is exposed is referred to as I_n_: I_1_ to I_4_ are recorded.

**9a. Circular sections:** the object is reconstructed assuming approximately circular cross-sections throughout. The method is applied using the iterations of exposure I_1_, I_2_, I_3_ and I_4_. In approximately circular cross-sections, as I_2_ is after or simultaneous to I_1_, volumes reconstructed from I_2_ are larger than I_1_-based volumes. Accordingly, I_4_ volumes replicate almost all of the original voxels and provide many redundant voxels while almost all voxels in I_1_ reconstructions are correctly placed, but many original model voxels are missed.

**9b. Elliptical sections:** ratios between iteration-of-exposure of different faces are used to derive the proportions of assumed elliptical cross-sections. Where elliptical ratio (the ratio of major to minor axes) is high, I_2_, I_3_ and I_4_ might be expected to be significantly later than I_1_. Therefore, a ratio I_n_ > 1 to I_1_ could replicate the elliptical ratio of the original cross-section. The ratios of I_n_ > 1: I_1_ and a mean (I_4_ + I_3_ + I_2_)/3: I_1_—are compared. The four aforementioned specimens don’t exhibit significant elliptical ratios. Instead, 411 skeleton voxels from five LRB root samples with varying elliptical ratios significantly greater than one are examined (“elliptical root samples” as described above)^3^. A linear function is derived from the correlation between elliptical ratio and each I_n_:I_1_ ratio, and applied to I_1_-I_4_ reconstructions—in I_1_ and I_2_ reconstructions, the major axis is enlarged, while the minor axes in I_3_ and I_4_ reconstructions are reduced.

### Assessment

Several reviews describe desirable characteristics of skeletonisation workflows^28, 29, 38, 41, 53^. In application to living architecture, some characteristics (centeredness, homotopy) are clearly more useful than others (smoothness, regularisation). In this study we settle on seven characteristics to assess our skeletonisation results: homotopy, skeleton thinness, skeleton centeredness, rotational invariance, volume reconstructibility, scalability (in computing time and data efficiency), and sample robustness (to noise and missing data), each of which is discussed below and covered in Tagliasacchi et al. and Cornea et al^28, 29^.

As stated by Arcelli et al.^45^, deleting only simple points ensures topology is preserved (*homotopy*). *Thinness* is assessed as containing no voxels that do not preserve topology and can be checked by deleting any non-curve-end voxels.

To assess *centeredness*, deviation from the PC centroid is compared between the present method and Huang’s^35^ L1-medial method at through-sectional positions in the four samples. Skeleton points within 0.5 voxel-widths of the PC centroids are called ‘correctly’ assigned, within 1.5 widths (adjacent voxel) are ‘acceptable’ and larger deviations are called ‘poor’. As in Arcelli et al.^45^, *volume reconstructibility* is given by the proportion of voxels in the original model reconstructed from the skeleton. Additionally, the proportion of reconstructed voxels that are correctly assigned is assessed. This is compared for four permutations (I_1_-I_4_) in assumed circular cross-section elements. The elliptical volumes reconstructed by the method described in step 9b are compared with circular I_1_ to I_4_ reconstructions.

*Scalability* is gauged by comparing the computation time and data reduction of samples of different original size^53^. Similar to Arcelli et al.^45^, data efficiency is given as a proportion of the source PC. *Sample robustness* of the thinning process (step 7) is assessed by comparing skeletons derived from noisy PCs. Pseudorandom noise in a Gaussian distribution with standard deviation proportional to the voxel size was added to the point coordinates of the four samples. Skeleton homotopy, centeredness, and processing time were inspected at a range of standard deviations. *Rotational invariance* is assessed here by comparing skeletons produced from identical models processed at 90° from one another. The skeleton centeredness and overlap of skeleton voxels are compared.

### Plant specimen collection

Appropriate permissions for photogrammetric survey were obtained for all specimens. The study involved no destruction, transplant, removal or damage of any plants.

### Plant use

The use of plants in this study complies with the Convention on Biological Diversity (Nagoya Protocol).

## Results

### Photogrammetric documentation

**Figure 4:**
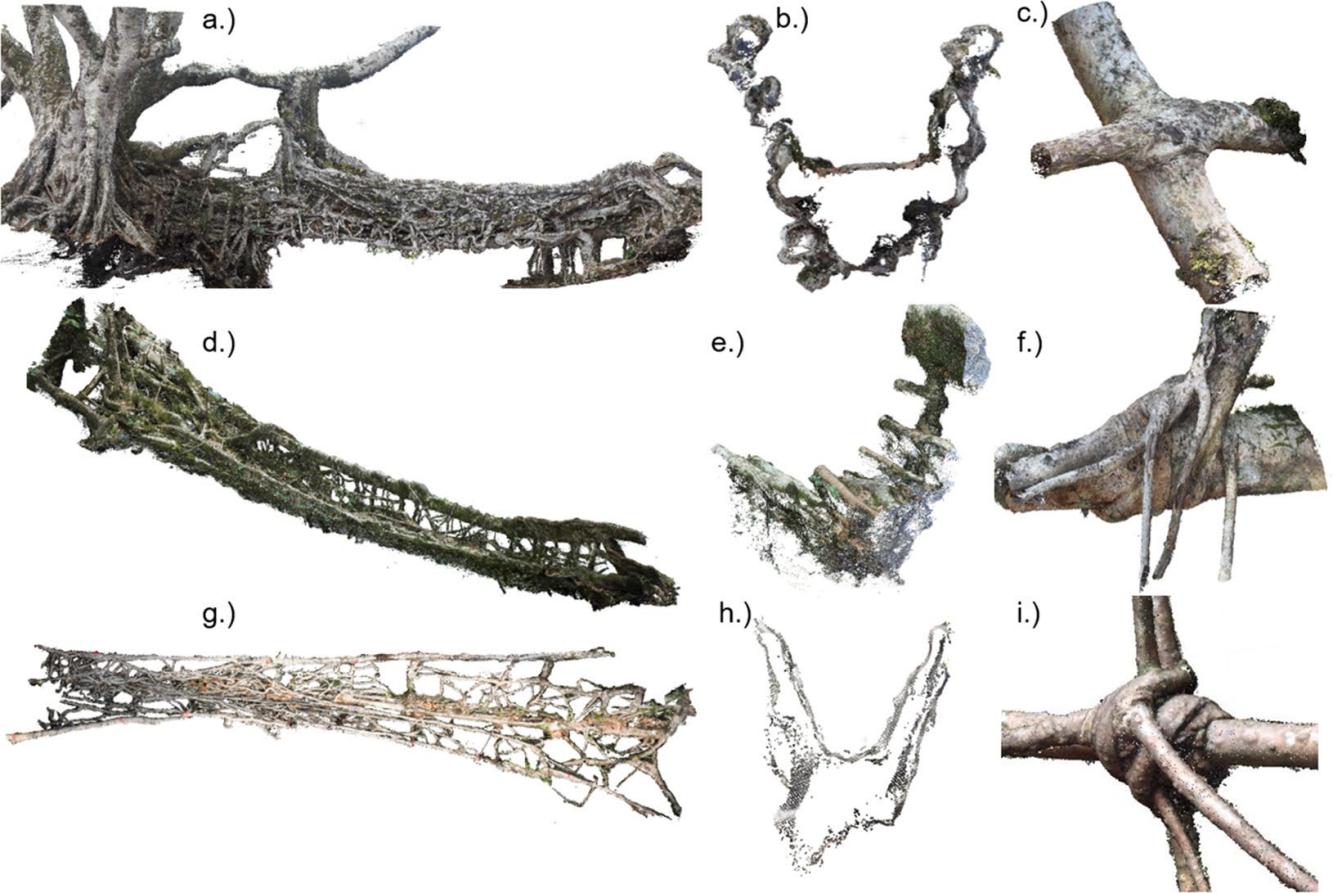
The photogrammetric surveys of living root bridges resulted in PCs of differing quality. (**a**) and (**b**) show the generally complete Nongbareh bridge; (**d**) and (**e**) show Niah Li bridge, relatively complete on the top, missing details on the underside; (**g**) shows the details captured on the top of Kudeng Rim bridge, missing perspectives from underneath - shown by areas of low point density in (**h**). (**c**), (**f**) and (**i**) are details from other bridges.

**Figure 5:**
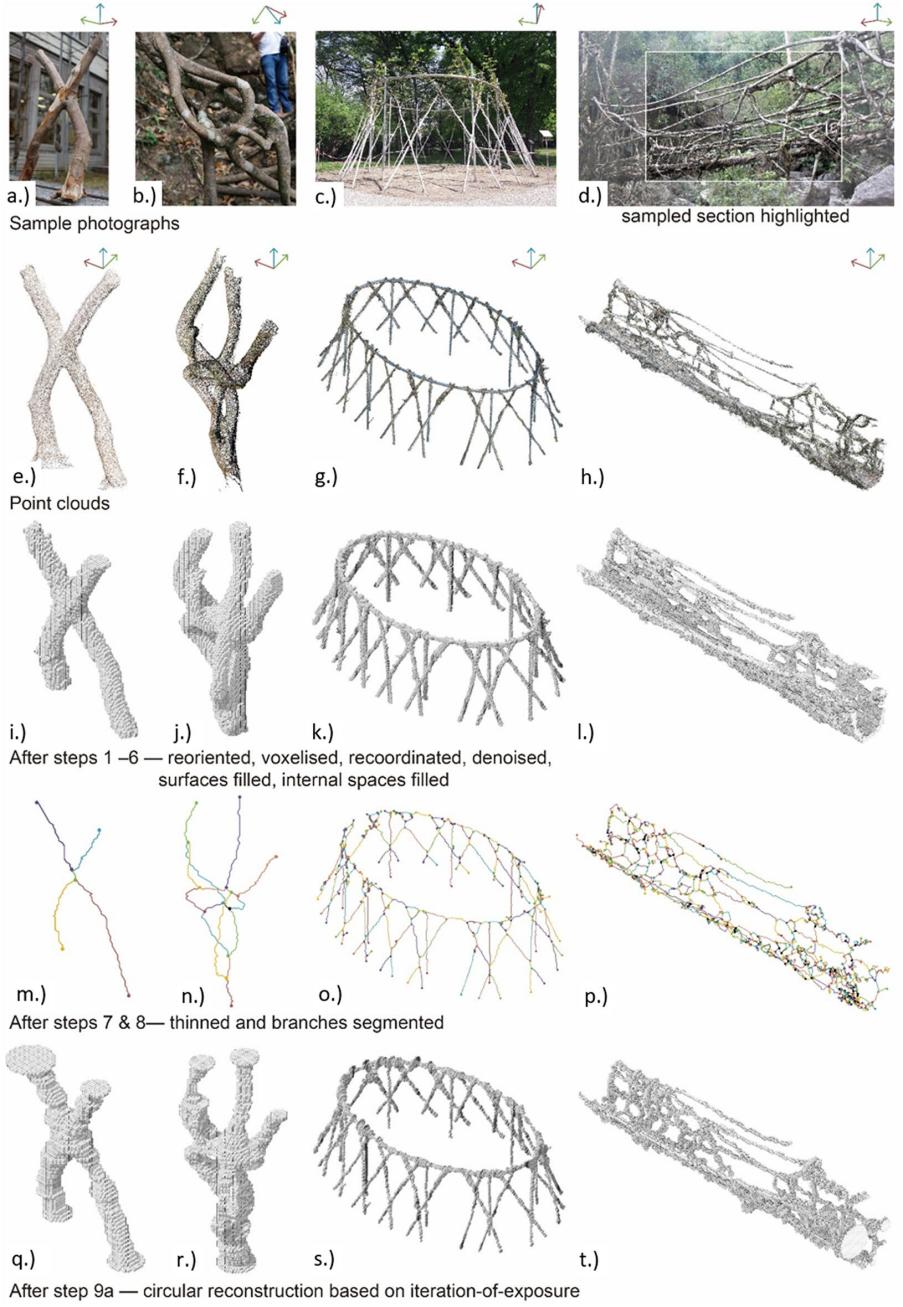
The Baubotanik joint, Ficus joint, Freiburg pavilion and Wah Koh La bridge as photographs (**a**–**d**) and corresponding point clouds (**e**–**h**); reoriented, voxelised model in Cartesian coordinates, denoised with any surface holes filled and the internal space filled (**i**–**l**); the resultant 1-voxel-thick skeleton, segmented by joint voxels (**m**–**p**); and circular volume reconstruction using iteration-of-exposure counts (**q**–**t**).

The photogrammetric surveys in Meghalaya generated 11 PCs of LRBs and five of specific details. The PCs generated vary in quality. Five LRB PCs are generally complete, with some or no details missing. These show the structurally important roots^3^ (shown in section in Fig. 4e) and their interconnections (Fig. 4d, g). Three PCs include all key perspectives, but are missing significant details. Three PCs are missing important perspectives (the bridge in Fig. 4a, b is complete; Fig. 4d, e is missing details; and Fig. 4g, h is missing important perspectives. Wah Koh La bridge is one of the three PCs missing important perspectives. Therefore it was chosen for the skeletonisation study to test the process’ robustness to sample quality. Incomplete PCs may be due to a combination of environmental problems (e.g. light dappling or nearby water), computational limits on number of photographs, and limited access to perspectives (e.g. below the deck in a canyon). The five PCs of details capture all key features—Fig. 4c, f, i. The stems of the Freiburg pavilion PC that resulted from the survey with the drone are generally complete (compare Fig. 5), with poor quality in the branches above the steel ring. The distortion in the bridge measured in 2018 and 2019 find an average deviation between the 2018 and 2019 surveys of 17 mm (standard deviation 19 mm). In the pavilion and two bridges using measurement points, the on-site and PC measurements match well. Average distortion, including photogrammetric distortions and measurement error, was 0.023% or 7 mm (standard deviation 4.5 mm), 0.52%, 12 mm (10 mm) and 0.12%, 17 mm (13 mm) in the pavilion and two bridges respectively. Wah Koh La bridge could not be scaled due to the missing perspectives in the PC.

### Skeletonisation

**Figure 6:**
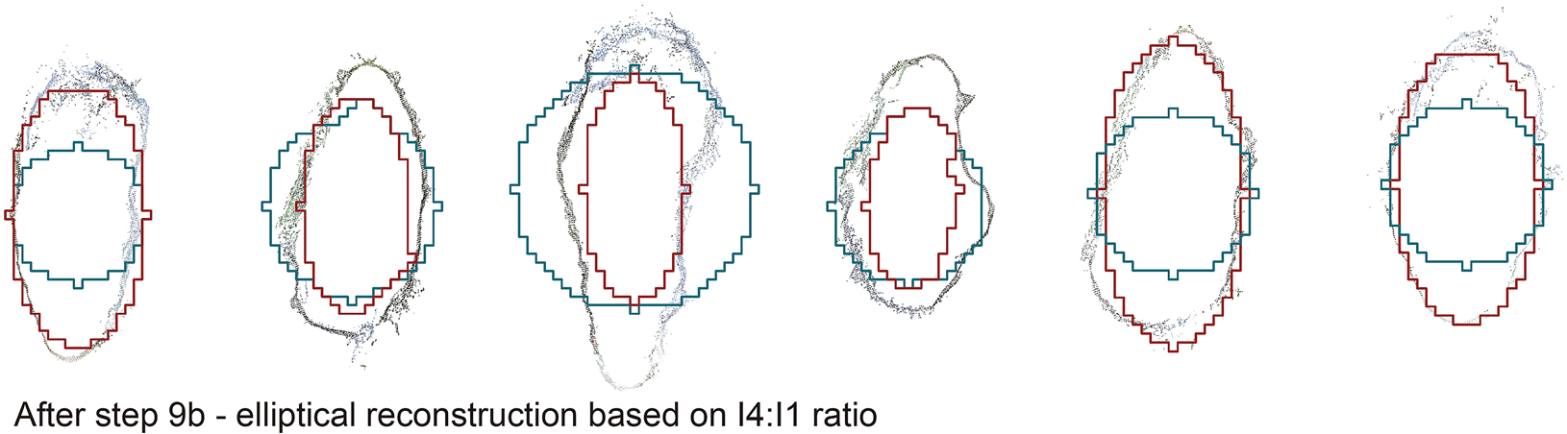
Examples of elliptical root sample PCs, reconstructed in step 9a as circles (blue) and 9b ellipses (red).

The outcomes of the workflow’s application to four example PCs (the Baubonanik joint, Ficus joint, Freiburg pavilion and Wah Koh La bridge) are presented in Figs. 5 and 6. Figure 5a–d shows example photos from the photogrammetric survey; Fig. 5e–h shows the input PCs; Fig. 5i–l show the oriented, voxelised model in Cartesian coordinates with noise excluded, surface holes filled and the internal space filled (steps 1 to 6); Fig. 5m–p show thin skeletons, segmented into “branches” (steps 7 & 8); Fig. 5q–t show the reconstructed circular-section volumes produced in step 9a; and Fig. 6 show cross-sections of elliptical roots, comparing circular and elliptical reconstructions with the original PC (step 9b).

The four samples differ in scale. The voxel size, defined in step 2, reflects the completeness and detail of the PC: more complete and detailed PCs allow for smaller voxels. The Baubotanik joint has 1.1 × 10^5^ approximately 7.5 mm object and void voxels, the Ficus joint 2.5 × 10^5^ approximately 7.4 mm voxels, the Freiburg pavilion 4.2 × 10^6^ 36 mm voxels, and the Wah Koh La bridge 6.2 × 10^5^ 58 mm voxels.

The skeleton extraction process is assessed against the seven characteristics described above—the findings are summarised in Table 1 and detailed below, in comparison with the L1-medial process^35^ applied to the same samples and a different voxel-thinning process, with data provided by Arcelli et al.^45^.

**Table 1:** Comparison of the present method, the L1-medial method applied to our PCs; and the findings of a previous study on a different voxel-thinning method.

Characteristic	Present method	L1-medial method, authors’ use of process from^35^	Distance-driven voxel method, data from^45^
Homotopy	Preserved through thinning process	Established after L1-medial	Preserved in distance-driven method
Thinness	1-Voxel-thick apart from at busy junctions	Busy junctions problem avoided in L1-medial	1-Voxel-thick apart from at busy junctions
Centeredness	87% within 1 voxel of centroid	80% within 1 voxel of centroid in L1-medial	Centeredness ensured before thinning
Volume reconstructibility	80–84% of original shape replicated	Established after L1-medial process	69% of original shape replicated
Scalability—computing time; data reduction	Computation time O(n) with n as voxel count; skeletons 0.56–2.7% of input object size	Computation time dependent on diverse variables; skeletons 0.08–1.3% of input object size	Computation time O(n) with n as voxel count; average skeleton 0.81% of input object size
Sample robustness	Robust to some noise and missing details	L1-medial robust to missing perspectives	Robust to some noise and missing details
Rotational invariance	Minor differences, other characteristics invariant	L1-medial is independent of rotation	Minor differences, other characteristics invariant

#### Homotopy

As described in the methods section, deletion criteria (a) and (b) ensure topology is preserved at every step because 26-connectedness is preserved for object voxels and 6-connectedness for void voxels. Both criteria (c) and (d) preserve many curve-end voxels, though not all possible situations are accounted for.

#### Thinness

A 1-voxel-thick skeleton is produced at all points apart from busy junctions where connectivity preservation is ensured^38^. In the skeletons shown, no object voxels can be deleted without breaking connectivity.

#### Centeredness

**Figure 7:**
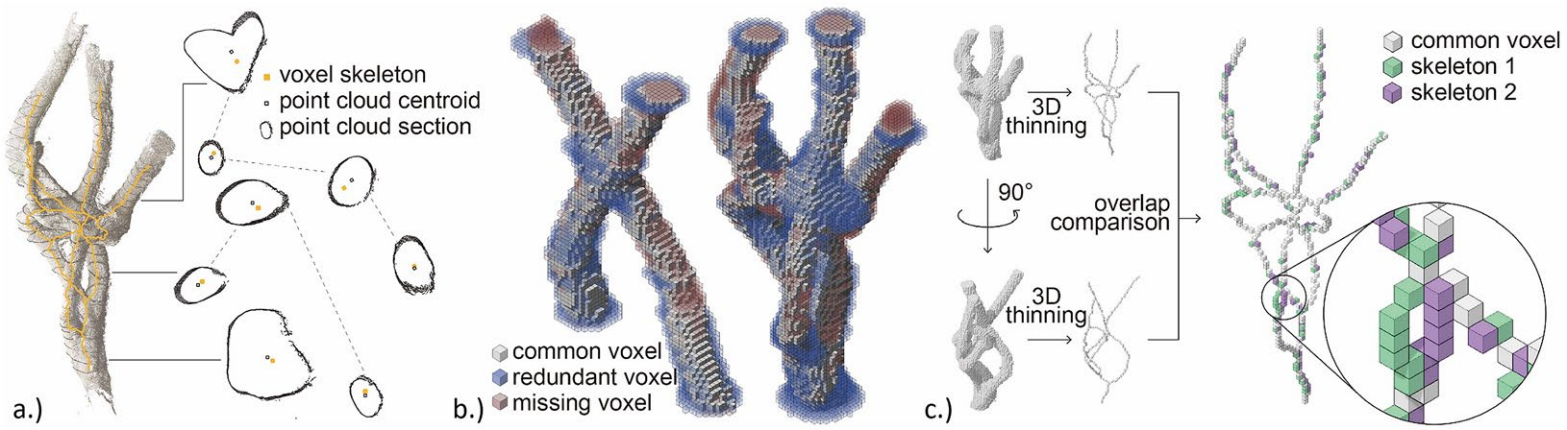
Comparison of skeleton centeredness with point cloud centroid at through sections of the Ficus joint (**a**); comparison of original and reconstructed voxel volumes in Baubotanik and Ficus joints (**b**); comparison of skeletons processed at different orientations (**c**).

Figure 7a shows the deviations between skeleton voxels and PC centroids. Deviation is counted in voxel-widths for 123 cross-sectional centroids in the Baubotanik joint (35), Ficus joint (53) and pavilion (35)—centroids could not be identified in the Wah Koh La sample. The problems relating to L1-medial methods resulted in five missing centroids (leaving 118 in total). Large deviations exist in both the present method (12.3% of measured points are more than 1.5 voxel widths from the centroid) and the L1-medial method (23%). 39% and 31% of voxels are correctly assigned (within 0.5 voxel widths) in the present and L1-medial methods respectively while 13% and 21% of voxels are more than 1.5 voxel-widths away, respectively.

#### Volume reconstructibility

Comparing I_1_ to I_4_ reconstructions, results are similar for each of the four samples: I_1_ and I_4_ reconstructions differ significantly from the original model and I_2_ in particular (and I_3_ to a lesser extent) reconstructions provide a high proportion of correct allocations and few redundant reconstructed voxels. Using I_2_ reconstruction, circular through-sections resulted in 84% replication of the original voxels in both the Baubotanik joint and Ficus joint. I_2_ reconstructions resulted in 49% and 41% replication in the pavilion and bridge samples respectively. This compares with 69% achieved in Arcelli’s method^45^. 77% and 73% of the reconstructed voxels were correctly allocated in the Baubotanik and Ficus joints respectively (i.e. 27% and 24% were wrongly allocated, respectively). In the pavilion and bridge, 77% and 66% were correctly allocated. Figure 7b shows the redundant reconstructed voxels (blue), missing original voxels (red) and common, correctly reproduced voxels (grey) in the Baubotanik and Ficus joints.

For the 411 elliptical cross-sections, a function of the ratio I_4_:I_1_ is found to best predict the true elliptical ratio (ER) of the section: the linear regression ER = 0.4255*(I_4_:I_1_) + 1.1235 has an R^2^ value of 0.4832. When applied to reconstructions based on I_1_ and I_4_, a high proportion of the original model voxels are replicated with few incorrect allocations, compared with I_2_ and I_3_. The elliptical reconstructions achieve a mean of 80% replication of original voxels and 76% correct voxels for I_1_ reconstructions, 33% more replication and just 9% more incorrect voxels than the mean I_1_ circular reconstruction.

#### Scalability

Computation time does not significantly increase with sample size for most steps. Steps 6 and 7 are the most computationally intensive. Step 6 took 90–23,577 s/Mb of input point cloud data (91–99.6% of total time) and step 7 took 8-109 s/Mb (0.5–8.1%) to compute. The major variance in computing time between samples is due to the dependence on the number of void voxels and the number of object voxels in steps 6 and 7 respectively. Huang’s process^35^ takes 19-88 s/Mb of input data, depending on a range of variables. Time needed for step 5, the manual hole filling, is defined initially by PC quality (i.e. the presence of holes), then by the sample’s complexity (in particular occlusions concealing holes) and voxel size (larger voxels cover holes). Due to these factors, hole filling took up to 10 min and 30 min in the Baubotanik and Ficus joints, respectively. In both the pavilion and bridge samples, no manual hole filling was needed because large voxels were used. The final skeletons are data light, irrespective of scale—the Baubotanik joint is reduced from 2.6 Mb in the original PC to 2.6 kb in the skeleton (0.1% of original size), the Ficus joint from 34 Mb to 7 kb (0.021%), the Freiburg pavilion from 80 Mb to 70 kb (0.088%) and the LRB from 74 Mb to 63 kb (0.085%). In Huang et al.^35^, skeletons were reduced to 0.08–1.3% of the original PC size and more complicated structures (with more joints and elements) produced larger skeletons.

#### Sample robustness

There are two major challenges presented by poor sampling: false topologies and skewed skeleton points. They are caused by PC surface holes and inaccurate (noisy) or imprecise (low resolution) surveys. These are mitigated by voxel size choice (step 2), density (step 4) and hole filling (step 5). Beyond this, step 7 provides some noise robustness. In the noisy PC tests, a robustness threshold was found around a standard deviation of noise at 0.6–0.75 times the voxel size. Below this, little or no false topologies occurred. In this range, the skeletons degraded significantly. Centeredness was generally unaffected apart from around the false topologies. Iteration count and processing time increase with noise. Within PCs, spatial resolution varies between areas: some perspectives are better captured than others. The voxel size is generally defined by low-resolution areas which can open surface holes—changes in resolution in the high- and mid-range areas are less relevant.

#### Rotational invariance

Voxels assigned to the skeleton change when the object is rotated. When rotated through 90°, 76% of the voxels in the Ficus joint are identical, and the maximum deviation between skeletons is 2 voxels, shown in Fig. 7c. The rotated skeleton is equally preserving of topology, equally thin, equally centred overall, and reconstructs the same proportion of original voxels (84%) with the same proportion of correct allocations (73%).

## Discussion

The results of the photogrammetric surveys show that detailed models of living architecture are achievable with OTS cameras and drones. However, details are more easily captured than whole bridges—complete PCs showing all perspectives of a complex bridge’s many details are difficult to achieve due to computing power, access, and lighting conditions. Lighting condition problems may be overcome with LiDAR scanning (with which the skeletonisation method is compatible), though instruments are currently too expensive for widespread use. Portable mobile mapping systems have recently developed significantly, with low-cost^54^ and high-accuracy^55^ options available. As these two benefits converge, future studies should investigate laser surveys’ benefits for living architecture. In comparison with previous documentation of simple length and width data of LRBs^3^, PCs provide much more detailed data, showing many of the key structural features of each bridge. In some cases, important sections are not visible due to self-occlusions but nonetheless can be partly inferred from visible parts by the PC viewer. Evaluating the scaling distortions, it is clear that photogrammetry can usually faithfully capture complex living architecture topologies but small distortions are to be expected, such as slightly miss-positioned elements. Such imprecisions could be avoided by integrating a step to compare PCs from different surveys before voxelisation. Comparing the specific details of photogrammetric surveys at different times allows change monitoring^46^. A necessary part of long-term documentation of living architecture is the interchangeability of tools—it should be possible to combine separate surveys. The results of this study and others show that environmental factors and accessibility are generally more significant than the differences between OTS cameras. Detailed models can be used for assessing tree health and growth remotely, for example by adapting the Visual Tree Assessment method^56^.

The presented workflow provides novel insight into the potential for iterative voxel-thinning in topological skeletonisation of living architecture specimens. As shown by the four examples, the method dependably reconstructs skeletons from complete or incomplete PCs and can exploit detailed PCs for precise skeletons. This offers an alternative to the L1-medial and distance-driven voxel methods. The proposed method shows better volume reconstruction than the distance-driven voxel method, particularly in smaller scale structures. The skeleton centeredness and homotopy show significant improvements in comparison with the L1-medial method. The results are otherwise comparable, apart from the computation and manual input time (the proposed method is significantly slower). While the method is robust to some noise, the skeleton quality degrades in very noisy PCs. Several aspects of the skeleton are balanced by the voxel size choice in step 2. Larger voxels reduce processing and manual input time and compensate for some noise, surface holes and low spatial resolution in the PC. In cases where these holes determine voxel size, the detail provided by high resolution areas is ‘wasted’. Small voxels improve the skeleton centeredness and could, in some cases, help preserve topology and make volume reconstruction more precise. The final skeleton is generally data light—each skeleton voxel contains three dimensional coordinates and three iteration counts: I_1_ and I_4_ (for elliptical ratios) and I_2_ (for circular reconstructions). This allows for flexible use in computer-aided design.

Potential future improvements lie in automation of the manual steps and in optimisation of the iteration-of-exposure reconstruction. Surface filling (step 5) is useful in combination with considered choice of octree depth (step 2). However, step 5 can be laborious. Automation of step 5 could be based on surface-patching^51^ if the missing surface is smooth. Step 2 could be automated with a persistent homology approach, in which the optimal voxel size is defined by topology preservation across scales^57^. In the iteration-of-exposure method there are three areas for improvement. Firstly, the reconstruction is in-plane, extrapolating in the x and y directions, forming circles/ellipses along the z axis. This differs from the thinning process, which is three dimensional. Future work could focus on 3D shape primaries. Secondly, voxels at modelling space boundaries and junctions take many iterations to erode, resulting in disproportionately wide reconstructed sections. Where unavoidable, this can be accounted for in the function linking iteration of exposure and elliptical ratio. Thirdly, each element is categorised by cross-section as circular or elliptical, neglecting deviations from these shape primaries (see Fig. 6). Future investigations could address other shapes such as the ‘inverted-T’ and ‘I’ shaped roots, as described by Ludwig et al.^3^ and Nicoll and Ray^6^. Skeletons with associated shape information can feed easily into the Euler–Bernoulli beam analysis employed by Jackson et al.^13^ and provides the topology of importance in James’ analysis^11^. The directional stiffness caused by elliptical elements can improve beam analyses. The full potential of axial elements in tree mechanics is still to be explored. For example, wood’s orthotropic properties are generally not considered^13^. Similar skeleton models are used for analyzing interactions occurring between plant components at different scales during plant growth simulations^9^. Our skeleton model can be translated into L-system languages (e.g. XL language in GroIMP^59^). In this way, it will open up further opportunities for studying resource allocation in relation to branching topology^58, 60^ of not just realistic but real plants. Beyond plants, the method could benefit analyses of anastomotic networks in biomedical fields including complex blood vessel maps^61^. By enabling mechanical and physiological analyses, a platform is offered for collaboration between arborists, engineers and architects.

As stated in the introduction, living architecture integrates physiology and mechanics and calls for an iterative design process^62^ in which regular documentation, modelling and analysis inform maintenance decisions. The workflow presented here provides a key part of this—structural analyses and growth predictions can be fed by a topologically correct skeleton, and in turn can feed decisions on root and shoot guidance or addition of technical elements. These decisions impact growth, which is once more documented using optical techniques.

## Conclusion

This study presents the first extensive 3D documentation of Meghalaya’s unique living root bridges and a method for meaningfully characterising the structure of these and other heritage and designed living architecture. The presented workflow uses low cost, off-the-shelf photogrammetric surveys to produce 1D skeletons via a process that recombines aspects of two previous voxel-thinning algorithms to provide centred, thin, topology-preserving, rotationally invariant skeletons for sections of living architecture. Associated shape information is preserved through a novel technique allowing element reconstruction. The skeletonisation process caters specifically to the challenges common in living architecture: anastomotic networks and diverse neighbouring elements. It includes steps for minimising problems induced by poor sampling. The method is applicable to small and large scale sections of differing complexity. By extracting the key topological features of a complex structure, the method provides a data-light, accurate representation that can be used in mechanical and physiological analyses and simulations of living structures in general. Thereby, the method facilitates design and analysis of growing structures, broadening designers’ horizons to complex forms of living architecture in high density urban contexts where precise predictive models are essential.

## Data Availability

The photogrammetric survey data and assessment data described in the current study are available from the corresponding author on reasonable request. The source code is available at: https://github.com/QiguanShu/skeleton-abstraction-of-point-cloud-by-voxel-thinning and the Freiburg pavilion, Ficus joint and Baubotanik joint PCs are available at: https://mediatum.ub.tum.de/1637267.

## References

[1] Arbona J, Greden L, Joachim M (2003). Nature's technology: The fab tree hab house. Thresholds.

[2] Graefe R (2014). Bauten aus lebenden Bäumen.

[3] Ludwig F, Middleton W, Gallenmüller F, Rogers P, Speck T (2019). Living bridges using aerial roots of ficus elastic—An interdisciplinary perspective. Sci. Rep..

[4] Höpfl L, Sunguroğlu Hensel D, Hensel M, Ludwig F (2021). Initiating research into adapting rural hedging techniques, hedge types, and hedgerow networks as novel urban green systems. Land.

[5] Middleton W, Habibi A, Shankar S, Ludwig F (2020). Characterizing regenerative aspects of living root bridges. Sustainability.

[6] Nicoll BC, Ray D (1996). Adaptive growth of tree root systems in response to wind action and site conditions. Tree Physiol..

[7] Ludwig F (2012). Botanische Grundlagen der Baubotanik und deren Anwendung im Entwurf.

[8] Ludwig, F., Wilfrid, M. & Vees, U. Baubotanik: Living Wood and Organic Joints. In *Rethinking Wood* (eds. Markus, P. & Hudert, S.) Ch. 5, 262–275 (Birkhäuser, 2019).

[9] Godin C, Costes E, Sinoquet H (1999). A method for describing plant architecture which integrates topology and geometry. Ann. Bot..

[10] Niklas KJ (1992). Plant Biomechanics: An Engineering Approach to Plant Form and Function.

[11] James KR, Haritos N, Ades PK (2006). Mechanical stability of trees under dynamic loads. Am. J. Bot..

[12] Müller U, Gindl W, Jeronimidis G (2006). Biomechanics of a branch–stem junction in softwood. Trees.

[13] Jackson T (2019). Finite element analysis of trees in the wind based on terrestrial laser scanning data. Agric. For. Meteorol..

[14] Fassi F, Achille C, Fregonese L (2011). Surveying and modelling the main spire of Milan Cathedral using multiple data sources. Photogram. Rec..

[15] Yilmaz HM, Yakar M, Gulec SA, Dulgerler ON (2007). Importance of digital close-range photogrammetry in documentation of cultural heritage. J. Cult. Herit..

[16] Omar H, Mahdjoubi L, Kheder G (2018). Towards an automated photogrammetry-based approach for monitoring and controlling construction site activities. Comput. Ind..

[17] Liang H (2018). The integration of terrestrial laser scanning and terrestrial and unmanned aerial vehicle digital photogrammetry for the documentation of Chinese classical gardens—A case study of Huanxiu Shanzhuang, Suzhou, China. J. Cult. Heritage.

[18] Luhmann T, Robson S, Kyle S, Boehm J (2019). Close-Range Photogrammetry and 3D Imaging.

[19] Surový P, Yoshimoto A, Panagiotidis D (2016). Accuracy of reconstruction of the tree stem surface using terrestrial close-range photogrammetry. Remote Sens..

[20] Forsman M, Börlin N, Holmgren J (2016). Estimation of tree stem attributes using terrestrial photogrammetry with a camera rig. Forests.

[21] Liang X (2014). The use of a hand-held camera for individual tree 3D mapping in forest sample plots. Remote Sens..

[22] Mokroš M (2018). High precision individual tree diameter and perimeter estimation from close-range photogrammetry. Forests.

[23] Hanke, K. & Moser, M. *Close-Range Photogrammetry and Laser Scanning Data Fusion and Complementary Approach for the Documentation of Complex Objects*. www.cipaheritagedocumentation.org (2011).

[24] Yoshinoa, K. & Okardab, B. Three-dimensional modeling of a tropical tree, melaleuca sp, using the digital photogrammetry. In *International Archives of the Photogrammetry, Remote Sensing and Spatial Information Sciences*, vol 38, 725–729 (2010).

[25] Quattrini, R., Malinverni, E. S., Clini, P., Nespeca, R. & Orlietti, E. From TLS to HBIM. High quality semantically-aware 3D modeling of complex architecture. In *International Archives of the Photogrammetry, Remote Sensing & Spatial Information Sciences,* 367–374 (2015).

[26] Hu S, Li Z, Zhang Z, He D, Wimmer M (2017). Efficient tree modeling from airborne LiDAR point clouds. Comput. Graph..

[27] Cheng Z-L, Zhang X-P, Chen B-Q (2007). Simple reconstruction of tree branches from a single range image. J. Comput. Sci. Technol..

[28] Tagliasacchi A, Delame T, Spagnuolo M, Amenta N, Telea A (2016). 3D skeletons: A state-of-the-art report. Comput. Graph. Forum.

[29] Cornea ND, Silver D, Min P (2007). Curve-skeleton properties, applications, and algorithms. IEEE Trans. Vis. Comput. Graph..

[30] Bucksch, A., Lindenbergh, R., Menenti, M. & Rahman, M. Z. In *Lidar Remote Sensing for Environmental Monitoring X* 746007 (International Society for Optics and Photonics).

[31] Verroust, A. & Lazarus, F. In *Proceedings Shape Modeling International'99. International Conference on Shape Modeling and Applications* 194–201 (IEEE, 1999).

[32] Wang Z (2014). A structure-aware global optimization method for reconstructing 3-D tree models from terrestrial laser scanning data. IEEE Trans. Geosci. Remote Sens..

[33] Xu H, Gossett N, Chen B (2007). Knowledge and heuristic-based modeling of laser-scanned trees. ACM Trans. Graph. (TOG).

[34] Bucksch A, Lindenbergh R (2008). CAMPINO—A skeletonization method for point cloud processing. ISPRS J. Photogramm. Remote. Sens..

[35] Huang H (2013). L1-medial skeleton of point cloud. ACM Trans. Graph..

[36] Su, Z., Li, S., Liu, H. & He, Z. Tree skeleton extraction from laser scanned points. In *IGARSS 2019–2019 IEEE International Geoscience and Remote Sensing Symposium* 6091–6094 (IEEE, 2019).

[37] Mei J, Zhang L, Wu S, Wang Z, Zhang L (2017). 3D tree modeling from incomplete point clouds via optimization and L1-MST. Int. J. Geogr. Inf. Sci..

[38] Saha PK, Borgefors G, di Baja GS (2016). A survey on skeletonization algorithms and their applications. Pattern Recognit. Lett..

[39] Manzanera, A., Bernard, T. M., Preteux, F. J. & Longuet, B. Unified mathematical framework for a compact and fully parallel nD skeletonization procedure. In *Vision Geometry VIII* 57–68 (International Society for Optics and Photonics, 1999).

[40] Ma CM, Sonka M (1996). A fully parallel 3D thinning algorithm and its applications. Comput. Vis. Image Underst..

[41] She, F. *et al.* In *2009 Digital Image Computing: Techniques and Applications* 14–18 (IEEE).

[42] Palágyi K, Kuba A (1999). A parallel 3D 12-subiteration thinning algorithm. Graph. Models Image Process..

[43] Subburaj K, Patil S, Ravi B (2006). Voxel-based thickness analysis of intricate objects. Int. J. CAD/CAM.

[44] Gorte B, Pfeifer N (2004). Structuring laser-scanned trees using 3D mathematical morphology. Int. Arch. Photogramm. Remote Sens..

[45] Arcelli C, di Baja GS, Serino L (2010). Distance-driven skeletonization in voxel images. IEEE Trans. Pattern Anal. Mach. Intell..

[46] Middleton W, Shu Q, Ludwig F (2019). Photogrammetry as a tool for living architecture. Int. Arch. Photogramm. Remote Sens. Spat. Inf. Sci..

[47] Agisoft Metashape Standard v. 1.5.1 (2019).

[48] Cloudcompare-open source project v. 2.11.3 (2011).

[49] Rusu, R. B. & Cousins, S. 3D is here: Point cloud library (pcl). In *2011 IEEE International Conference on Robotics and Automation* 1–4 (IEEE, 2011).

[50] Holland SM (2008). Principal Components Analysis (PCA).

[51] Attene M, Campen M, Kobbelt L (2013). Polygon mesh repairing: An application perspective. ACM Comput. Surv. (CSUR).

[52] Lohou C, Bertrand G (2004). A 3D 12-subiteration thinning algorithm based on P-simple points. Discrete Appl. Math..

[53] Sobiecki A, Jalba A, Telea A (2014). Comparison of curve and surface skeletonization methods for voxel shapes. Pattern Recognit. Lett..

[54] Luetzenburg G, Kroon A, Bjørk AA (2021). Evaluation of the Apple iPhone 12 Pro LiDAR for an application in geosciences. Sci. Rep..

[55] Barba, S., Ferreyra, C., Cotella, V. A., di Filippo, A. & Amalfitano, S. A SLAM integrated approach for digital heritage documentation. In *International Conference on Human–Computer Interaction* 27–39 (2021).

[56] Mattheck C, Breloer H (1994). Field guide for visual tree assessment (VTA). Arboricult. J..

[57] Li M, Duncan K, Topp CN, Chitwood DH (2017). Persistent homology and the branching topologies of plants. Am. J. Bot..

[58] Da Silva D, Favreau R, Auzmendi I, DeJong TM (2011). Linking water stress effects on carbon partitioning by introducing a xylem circuit into L-PEACH. Ann. Bot..

[59] Kniemeyer, O. & Kurth, W. The modelling platform GroIMP and the programming language XL. In *International Symposium on Applications of Graph Transformations with Industrial Relevance* 570–572 (2007).

[60] Merklein, J., Poirier-Pocovi, M., Buck-Sorlin, G. H., Kurth, W. & Long, Q. A dynamic model of xylem and phloem flux in an apple branch. In *2018 6th International Symposium on Plant Growth Modeling, Simulation, Visualization and Applications (PMA)* 50–55 (IEEE, 2018).

[61] Münzer B, Schoeffmann K, Böszörmenyi L (2018). Content-based processing and analysis of endoscopic images and videos: A survey. Multimed. Tools Appl..

[62] Shu Q, Middleton W, Dörstelmann M, Santucci D, Ludwig F (2020). Urban microclimate canopy: Design, manufacture, installation, and growth simulation of a living architecture prototype. Sustainability.

